# A Flexible Proof Format for SAT Solver-Elaborator Communication

**DOI:** 10.1007/978-3-030-72016-2_4

**Published:** 2021-03-01

**Authors:** Seulkee Baek, Mario Carneiro, Marijn J. H. Heule

**Affiliations:** 8 Eindhoven University of Technology, Eindhoven, The Netherlands; 9 Aalborg University, Aalborg East, Denmark; Carnegie Mellon University, Pittsburgh, PA United States

**Keywords:** Satisfiability, Proof format, DRAT, LRAT, FRAT

## Abstract

We introduce FRAT, a new proof format for unsatisfiable SAT problems, and its associated toolchain. Compared to DRAT, the FRAT format allows solvers to include more information in proofs to reduce the computational cost of subsequent elaboration to LRAT. The format is easy to parse forward and backward, and it is extensible to future proof methods. The provision of optional proof steps allows SAT solver developers to balance implementation effort against elaboration time, with little to no overhead on solver time. We benchmark our FRAT toolchain against a comparable DRAT toolchain and confirm >84% median reduction in elaboration time and >94% median decrease in peak memory usage.

## 1 Introduction

The *Boolean satsifiability problem* is the problem of determining, for a given Boolean formula consisting of Boolean variables and connectives, whether there exists a variable assignment under which the formula evaluates to true. Boolean satisfiability (SAT) is interesting in part because there are surprisingly diverse types of problems that can be encoded as Boolean formulas and solved efficiently by checking their satisfiability. *SAT solvers*, programs that automatically solve SAT problems, have been successfully applied to a wide range of areas, including hardware verification [2], planning [14], and combinatorics [12].

The performance of SAT solvers has taken great strides in recent years, and modern solvers can often solve problems involving millions of variables and clauses, which would have been unthinkable a mere 20 years ago [15]. But this improvement comes at the cost of significant increase in the code complexity of SAT solvers, which makes it difficult to either assume their correctness on faith, or certify their program correctness directly. As a result, the ability of SAT solvers to produce independently verifiable certificates has become a pressing necessity. Since there is an obvious certificate format (the satisfying boolean assignment) for satisfiable problems, the real challenge in proof-producing SAT

* Partially supported by AFOSR grant FA9550-18-1-0120
** Supported by the National Science Foundation under grant CCF-2010951

solving is in devising a compact proof format for unsatisfiable problems, and developing a toolchain that efficiently produces and verifies it.

The current de facto standard proof format for unsatisfiable SAT problems is DRAT [10]. The format, as well as its predecessor DRUP, were designed with a strong focus on quick adaptation by the community, emphasizing easy proof emission, practically zero overhead, and reasonable validation speed [11]. The DRAT format has become the only supported proof format in SAT Competition and Races since 2014 due to entrants losing interest in alternatives.

DRAT is a *clausal* proof format [6], which means that a DRAT proof consists of a sequence of instructions for adding and deleting clauses. It is helpful to think of a DRAT proof as a program for modifying the 'active multiset' of clauses: the initial active multiset is the clauses of the input problem, and this multiset grows and shrinks over time as the program is executed step by step. The invariant throughout program execution is that the active multiset at any point of time is *at least as satisfiable* as the initial active multiset. This invariant holds trivially in the beginning and after a deletion; it is also preserved by addition steps by either RUP or RAT, which we explain shortly. The last step of a DRAT proof is the addition of the empty clause, which ensures the unsatisfiability of the final active multiset, and hence that of the initial active multiset, i.e. the input problem.

Every addition step in DRAT is either a *reverse unit propagation* (RUP) step [6] or a *resolution asymmetric tautology* (RAT) [13] step. A clause $C$ has the property AT (asymmetric tautology) with respect to a formula $F$ if $F, \overline{C} \vdash_1 \bot$, which is to say, there is a proof of the empty clause by unit propagation using $F$ and the negated literals in $C$. A RUP step that adds $C$ to the active multiset $F$ is valid if $C$ has property AT with respect to $F$. A clause $l \vee C$ has property RAT with respect to $F$ if for every clause $\bar{l} \vee D \in F$, the clause $C \vee D$ has property AT with respect to $F$. In this case, $C$ is not logically entailed by $F$, but $F$ and $F \wedge C$ are equisatisfiable, and a RAT step will add $C$ to the active multiset if $C$ has property RAT with respect to $F$. (See [10] for more about the justification for this proof system.)

DRAT has a number of advantages over formats based on more traditional proof calculi, such as resolution or analytic tableaux. For SAT solvers, DRAT proofs are easier to emit because CNF clauses are the native data structures that the solvers store and manipulate internally. Whenever a solver obtains a new clause, the clause can be simply streamed out to a proof file without any further modification. Also, DRAT proofs are more compact than resolution proofs, as the latter can become infeasibly large for some classes of SAT problems [7].

There is, however, room for further improvement in the DRAT format due to the information loss incurred by DRAT proofs. Consider, for instance, the SAT problem and proofs shown in Figure 1. The left column is the input problem in the DIMACS format, the center column is its DRAT proof, and the right column is the equivalent proof in the LRAT format, which can be thought of as an enriched version of DRAT with more information. The numbers before the first zero on lines without a "`d`" represent literals: positive numbers denote positive literals, while negative numbers denote negative literals. The first clause of the input formula is $(x_1 \vee x_2 \vee \overline{x}_3)$, or equivalently `1 2 -3 0` in DIMACS.

The first lines of both DRAT and LRAT proofs are RUP steps for adding the clause $(x_1 \vee x_2)$, written `1 2 0`. When an LRAT checker verifies this step, it is informed of the IDs of active clauses (the trailing numbers `1 6 3`) relevant for unit propagation, in the exact order they should be used. Therefore, the LRAT checker only has to visit the first, sixth, and third clauses and confirm that, starting with unit literals $\overline{x_1}, \overline{x_2}$, they yield the new unit literals $\overline{x_3}, x_4, \bot$. In contrast, a DRAT checker verifying the same step must add the literals $\overline{x_1}, \overline{x_2}$ to the active multiset (in this case, the eight initial clauses) and carry out a blind unit propagation with the whole resulting multiset until contradiction. This omission of RUP information in DRAT proofs introduces significant overheads in proof verification. Although the exact figures vary from problem to problem, checking a DRAT proof typically takes approximately twice as long as solving the original problem, whereas the verification time for an LRAT proof is negligible compared to its solution time. This additional cost of checking DRAT proofs also represents a lost opportunity: when a SAT solver emits a RUP step, it knows exactly how the new clause was obtained, and this knowledge can (in theory) be turned into an LRAT-style RUP annotation, which can cut down verification costs significantly if conveyed to the verifier.

For the DRAT format, a design choice was made not to include such information since demanding explicit proofs for all steps turned out to be impractical. Although it is *theoretically* possible to always glean the correct RUP annotation from the solver state, computing this information can be intricate and costly for some types of inferences (e.g. conflict-clause minimization [22]), making it harder to support proof logging [25]. Reducing such overheads is particularly important for solving satisfiable formulas, as proofs are superfluous for them and the penalty for maintaining such proofs should be minimized. We should note, however, that proof elaboration need not be an all-or-nothing business; if it is infeasible to demand 100% elaborated proofs, we can still ask solvers to fill in as many gaps as it is convenient for them to do so, which would still be a considerable improvement over handling all of it from the verifier side.

Inclusion of final clauses is another potential area for improvement over the DRAT format. A DRAT proof typically includes many addition steps that do not ultimately contribute to the derivation of the empty clause. This is unavoidable in the proof emission phase, since a SAT solver cannot know in advance whether a given clause will be ultimately useful, and must stream out the clause before it can find out. All such steps, however, should be dropped in the post-processing phase in order to compress proofs and speed up verification. The most straightforward way of doing this is processing the proof in reverse order [6]: when processing a clause $C_{k+1}$, identify all the clauses used to derive $C_{k+1}$, mark them as 'used', and move on to clause $C_k$. For each clause, process it if it is marked as used, and skip it otherwise. The only caveat of this method is that the postprocessor needs to know which clauses were present at the very end of the proof, since there is no way to identify which clauses were used to derive the

```
   DIMACS             DRAT                    LRAT

p cnf 4 8              1 2 0        9 1 2 0        1 6 3 0
 1  2 -3 0      d  1 -3 2 0         9                 d 1 0
-1 -2  3 0             1 3 0       10 1 3 0        9 8 6 0
 2  3 -4 0      d   1 4 3 0        10                 d 6 0
-2 -3  4 0               1 0       11   1 0     10 9 4 8 0
-1 -3 -4 0      d      1 3 0       11           d 10
 1  3  4 0      d      1 2 0                          9
-1  2  4 0      d 1 -4 -2 0                             8 0
 1 -2 -4 0               2 0       12   2 0     11 7 5 3 0
                d  -1 4 2 0        12               d 7
                d  2 -4 3 0                             3 0
                           0       13     0 11 12 2 4 5 0
```

**Fig. 1.** DRAT and LRAT proofs of a SAT problem. All whitespace and alignment is not significant; we have aligned lines of the DRAT proof with the corresponding LRAT lines (`d` steps in LRAT may correspond to multiple DRAT `d` steps).

empty clause otherwise. Although it is possible to enumerate the final clauses by a preliminary forward pass through a DRAT proof, this is clearly unnecessary work since SAT solvers know exactly which clauses are present at the end, and it is desirable to put this information in the proof in the first place.

## 2 The FRAT format

To address the above issues, we introduce FRAT, a new proof format designed to allow fine-grained communication between SAT solvers and elaborators. The main differences between FRAT and DRAT are:

(1) optional annotation of RUP steps,
(2) inclusion of final clauses, and
(3) identification of clauses by unique IDs.

We've already explained the rationale for (1) and (2); (3) is necessary for concise references to clauses in deletions and RUP step annotations. More specifically, a FRAT proof consists of the following six types of proof steps:

- `o`: An original step; a clause from the input file. The purpose of these lines is to name the clauses from the input with identifiers; they are not required to come in the same order as the file, they are not required to be numbered in order, and not all steps in the input need appear here. Proof may also progress (with `a` and `d` steps) before all `o` steps are added.
- `a`, `l`: An addition step, and an optional LRAT-style unit propagation proof of the step. The proof, if provided, is a sequence of clauses in the current formula in the order that they become unit. For solver flexibility, they are allowed to come out of order, but the elaborator is optimized for the case where they are correctly ordered. For a RAT step, the negative numbers in the proof refer to the clauses in the active set that contain the negated pivot literal, followed by the unit propagation proof of the resolvent. See [3] for more details on the LRAT checking algorithm.

`d`: A deletion step for deleting the clause with the given ID from the formula. The literals given must match the literals in the corresponding addition step up to permutation.

`r`: A relocation step. The syntax is `r` $\langle ids \rangle$ `0`, where $\langle ids \rangle$ has the form $s_0$, $t_0$, $\ldots$, $s_k$, $t_k$ and must consist of an even number of clause IDs. It indicates that the active clause with ID $s_i$ is re-labeled and now has ID $t_i$, for each $0 \le i \le k$. (This is used for solvers that use pointer identity for clauses, but also do garbage collection to decrease memory fragmentation.)

`f`: A finalization step. These steps come at the end of a proof, and provide the list of all active clauses at the end of the proof. The clauses may come in any order, but every step that has been added and not deleted must be present. (For best results, clauses should be finalized in roughly reverse order of when they were added.)

(Our modified version of CaDiCaL also outputs a seventh kind of step, `t` $\langle todo\_id \rangle$ `0`, to collect statistics on code paths that produce `a` steps without proofs. See Section 3 for how this information is used.)

Figure 1 is an example from [3], which includes a SAT problem in DIMACS format, and the proofs of its unsatisfiability in DRAT and LRAT formats. It shows how proofs are produced and elaborated via the DRAT toolchain. Figure 2 shows the corresponding problem and proofs for the FRAT toolchain. Notice how the FRAT proof is more verbose than its DRAT counterpart and includes all the hints for addition steps, which are reused in the subsequent LRAT proof.

**Binary FRAT** The files shown in Figure 2 are in the text version of the FRAT format, but for efficiency reasons solvers may also wish to use a binary encoding. The binary FRAT format is exactly the same in structure, but the integers are encoded using the same variable-length integer encoding used in binary DRAT [9]. Unsigned numbers are encoded in 7-bit little endian, with the high bit set on each byte except the last. That is, the number

$$n = x_0 + 2^7 x_1 + \cdots + 2^{7k} x_k$$

(with each $x_i < 2^7$) is encoded as

$$\mathtt{1}x_0\ \mathtt{1}x_1\ \ldots\ \mathtt{0}x_k.$$

Signed numbers are encoded by mapping $n \geq 0$ to $f(n) := 2n$ and $-n$ (with $n > 0$) to $f(n) := 2n + 1$, and then using the unsigned encoding. (Incidentally, the mapping $f$ is not surjective, as it misses 1. But it is used by other formats so we have decided not to change it.)

FRAT

```
o 1                 1 2 -3 0  |  f 1    1 2 -3 0
o 2               -1 -2 3 0   |  f 2   -2 -1 3 0
o 3                 2 3 -4 0  |  f 3    2 3 -4 0
o 4               -2 -3 4 0   |  f 4   -2 -3 4 0
o 5              -1 -3 -4 0   |  f 5 -1 -3 -4 0
o 6                  1 3 4 0  |  f 6     1 3 4 0
o 7                -1 2 4 0   |  f 7    -1 2 4 0
o 8               1 -2 -4 0   |  f 8  1 -2 -4 0
a 9 -3 -4 0 l        5 1 8 0  |  f 9     -3 -4 0
a 10   -4 0 l     9 3 2 8 0   |  f 10        -4 0
a 11    3 0                   |  f 11         3 0
a 12   -2 0                   |  f 12        -2 0
a 13    1 0 l     12 11 1 0   |  f 13         1 0
a 14      0 l 13 12 10 7 0    |  f 14           0
```

LRAT

```
 9 -3 -4 0       5 1 8 0
 9                 d 5 0
10    -4 0     9 3 2 8 0
10             d 8 3 9 0
11     3 0    10 6 7 2 0
11               d 2 6 0
12    -2 0      11 10 4 0
12                 d 4 0
13     1 0      12 11 1 0
13              d 1 11 0
14       0 13 12 10 7 0
```

**Fig. 2.** FRAT and LRAT proofs of a SAT problem. To illustrate that proofs are optional, we have omitted the proofs of steps 11 and 12 in this example. The steps must still be legal RAT steps but the elaborator will derive the proof rather than the solver.

## 2.1 Flexibility and extensibility

The purpose of the FRAT format is for solvers to be able to quickly write down what they are doing while they are doing it, with the elaborator stage "picking up the pieces" and preparing the proof for consumption by simpler mechanisms such as certified LRAT checkers. As such, it is important that we are able to concisely represent all manner of proof methods used by modern SAT solvers.

The high level syntax of a FRAT file is quite simple: A sequence of "segments", each of which begins with a character, followed by zero or more nonzero numbers, followed by a `0`. In the binary version, each segment similarly begins with a printable character, followed by zero or more nonzero bytes, followed by a zero byte. (Note that continuation bytes in an unsigned number encoding are always nonzero.) This means that it is possible to jump into a FRAT file and find segment boundaries by searching for a nearby zero byte.

$$
\begin{aligned}
\langle proof \rangle &\leftarrow \langle line \rangle^* \\
\langle line \rangle &\leftarrow \langle orig \rangle \mid \langle add \rangle \mid \langle del \rangle \mid \langle final \rangle \mid \langle reloc \rangle \\
\langle add \rangle &\leftarrow \langle add\_seg \rangle \mid \langle add\_seg \rangle\ \langle hint \rangle \\
\langle orig \rangle &\leftarrow \texttt{o}\ \langle id \rangle\ \langle literal \rangle^*\ \texttt{0} \\
\langle add\_seg \rangle &\leftarrow \texttt{a}\ \langle id \rangle\ \langle literal \rangle^*\ \texttt{0} \\
\langle del \rangle &\leftarrow \texttt{d}\ \langle id \rangle\ \langle literal \rangle^*\ \texttt{0} \\
\langle final \rangle &\leftarrow \texttt{f}\ \langle id \rangle\ \langle literal \rangle^*\ \texttt{0} \\
\langle reloc \rangle &\leftarrow \texttt{r}\ (\langle id \rangle\ \langle id \rangle)^*\ \texttt{0} \\
\langle hint \rangle &\leftarrow \texttt{l}\ (\langle id \rangle \mid -\langle id \rangle)^*\ \texttt{0} \\
\langle id \rangle &\leftarrow \langle pos \rangle \\
\langle literal \rangle &\leftarrow \langle pos \rangle \mid \langle neg \rangle \\
\langle neg \rangle &\leftarrow -\langle pos \rangle \\
\langle pos \rangle &\leftarrow [\texttt{1-9}]\ [\texttt{0-9}]^*
\end{aligned}
$$

**Fig. 3.** Context-free grammar for the FRAT format.

| | | | | | | | | | | |
|---|---|---|---|---|---|---|---|---|---|---|
| text | a | 9 | -3 | -4 | 0 | l | 5 | 1 | 8 | 0 |
| binary | 61 | 09 | 07 | 09 | 00 | 6C | 0A | 02 | 10 | 00 |

**Fig. 4.** Comparison of binary and text formats for a step. Note that the step ID 9 uses the unsigned encoding, but literals and LRAT style proof steps use signed encoding.

This is in contrast to binary LRAT, in which add steps are encoded as `a` $\langle id \rangle$ $\langle literal \rangle^*$`0` $(\pm\langle id \rangle)^*$ `0`, because a random zero byte could either be the end of a segment or the middle of an add step. Since `0x61`, the ASCII representation of `a`, is also a valid step ID (encoding the signed number $-48$), in a sequence such as (`a` $\langle nonzero \rangle^*$ `0`)$^*$, the literals and the steps cannot be locally disambiguated.

The local disambiguation property is important for our FRAT elaborator, because it means that we can efficiently parse FRAT files generated by solvers *backward*, reading the segments in reverse order so that we can perform backward checking in a single pass.

DRAT is based on adding clauses that are RAT with respect to the active formula. It is quite versatile and sufficient for most common cases, covering CDCL steps, hyper-resolution, unit propagation, blocked clause elimination and many other techniques. However, we recognize that not all methods can be cast into this format, or are too expensive to translate into this proof system. In this work we define only six segment characters (`a`, `d`, `f`, `l`, `o`, `r`), that suffice to cover methods used by SAT solvers targeting DRAT. However, the format is forward-compatible with new kinds of proof steps, that can be indicated with different characters.

For example, CryptoMiniSat [21] is a SAT solver that also supports XOR clause extraction and reasoning, and can derive new XOR clauses using proof techniques such as Gaussian elimination. Encoding this in DRAT is quite complicated: The XOR clauses must be Tseitin transformed into CNF, and Gaussian elimination requires a long resolution proof. Participants in SAT competitions therefore turn this reasoning method off as producing the DRAT proofs is either too difficult or the performance gains are canceled out by the overhead.

FRAT resolves this impasse by allowing the solver to express itself with minimal encoding overhead. A hypothetical extension to FRAT would add new segment characters to allow adding and deleting XOR clauses, and a new proof method for proof by linear algebra on these clauses. The FRAT elaborator would be extended to support the new step kinds, and it could either perform the expensive translation into DRAT at that stage (only doing the work when it is known to be needed for the final proof), or it could pass the new methods on to some XLRAT backend format that understands these steps natively. Since the extension is backward compatible, it can be done without impacting any other FRAT-producing solvers.

## 3 FRAT-producing solvers

The FRAT proof format is designed to allow conversion of DRAT-producing solvers into FRAT-producing solvers at minimal cost, both in terms of implementation effort and impact on runtime efficiency. In order to show the feasibility of such conversions, we chose two popular SAT solvers, CaDiCaL[1] and MiniSat[2], to modify as case studies. The solvers were chosen to demonstrate two different aspects of feasibility: since MiniSat forms the basis of the majority of modern SAT solvers, an implementation using MiniSat shows that the format is widely applicable, and provides code which developers can easily incorporate into a large number of existing solvers. CaDiCaL, on the other hand, is a cutting-edge modern solver which employs a wide range of sophisticated optimizations. A successful conversion of CaDiCaL shows that the technology is scalable, and is not limited to simpler toy examples.

As mentioned in Section 2, the main solver modifications required for FRAT production are inclusions of clause IDs, finalization steps, and LRAT proof traces. The provision of IDs requires some non-trivial modification as many solvers, including CaDiCaL and MiniSat, do not natively keep track of clause IDs, and DRAT proofs use literal lists up to permutation for clause identity. In CaDiCaL, we added IDs to all clauses, leading to 8 bytes overhead per clause. Additionally, unit clauses are tracked separately, and ensuring proper ID tracking for unit clauses resulted in some added code complexity. In MiniSat, we achieved 0 byte overhead by using the pointer value of clauses as their ID, with unit clauses having computed IDs based on the literal. This requires the use of relocation steps during garbage collection. The output of finalization steps requires identifying

[1] https://github.com/digama0/cadical
[2] https://github.com/digama0/minisat the active set from the solver state, which can be subtle depending on the solver architecture, but is otherwise a trivial task assuming knowledge of the solver.

LRAT trace production is the heart of the work, and requires the solver to justify each addition step. This modification is relatively easier to apply to MiniSat, as it only adds clauses in a few places, and already tracks the "reasons" for each literal in the current assignment, which makes the proof trace straightforward. In contrast, CaDiCaL has over 30 ways to add clauses; in addition to the main CDCL loop, there are various in-processing and optimization passes that can create new clauses.

To accommodate this complexity, we leverage the flexibility of the FRAT format which allows optional hints to focus on the most common clause addition steps, to reap the majority of runtime advantage with only a few changes. The FRAT elaborator falls back on the standard elaboration-by-unit propagation when proofs are not provided, so future work can add more proofs to CaDiCaL without any changes to the toolchain.

To maximize the efficacy of the modification, we used a simple method to find places to add proofs. In the first pass, we added support for clause ID tracking and finalization, and changing the output format to FRAT syntax. Since CaDiCaL was already producing DRAT proofs, we can easily identify the addition and removal steps and replace them with `a` and `d` steps. Once this is done, CaDiCaL is producing valid FRAT files which can pass through the elaborator and get LRAT results, but it will be quite slow since the FRAT elaborator is essentially acting as a less-optimized version of DRAT-trim at this point.

We then find all code paths that lead to an `a` step being emitted, and add an extra call to output a step of the form `t` $\langle todo\_id \rangle$ `0`, where $\langle todo\_id \rangle$ is some unique identifier of this position in the code. The FRAT elaborator is configured to ignore these steps, so they have no effect, but by running the solver on benchmarks we can count how many `t` steps of each kind appear, and so see which code paths are hottest.

The basic idea is that elaborating a step that has a proof is much faster than elaborating a step that doesn't, but the distribution of code paths leading to add steps is highly skewed, so adding proofs to to the top 3 or 4 paths already decreases the elaboration time by over 70%. At the time of writing, about one third of CaDiCaL code paths are covered, and median elaboration time is about 15% that of DRAT-trim (see Section 5). (This is despite the fact that our elaborator could stand to improve on low level optimizations, and runs about twice as slow as DRAT-trim when no proofs are provided.)

## 4 Elaboration

The main tasks of the FRAT-to-LRAT elaborator[3] are provision of missing RUP step hints, elimination of irrelevant clause additions, and re-labeling clauses with new IDs. These tasks are performed in two separate 'passes' over files, writing

[3] The elaborator used for this paper can be found at https://github.com/digama0/frat/tree/tacas.

**Algorithm 1** First pass (elaboration): FRAT to elaborated reversed FRAT

```
function Elaborate(cert)
    F ← ∅, revcert ← []          ▷ F is a map ID → clause with a bool marking
    for step in reverse(cert) do
        case step of
            o(i, C) ⇒
                C′ ← F.remove(i); assert C′ ≃ C
                if C′.marked then revcert ← revcert, o(i, C)
            a(i, C, proof?) ⇒
                C′ ← F.remove(i); assert C′ ≃ C
                if C′.marked then
                    steps′ ← case proof? of
                        ε ⇒ ProveRAT(F, C)
                        l(steps) ⇒ CheckHint(F, C, steps)
                    for j in {j | ±j ∈ steps′} do
                        if ¬F_j.marked then
                            F_j.marked ← true
                            revcert ← revcert, d(step, F_j)
                    revcert ← revcert, a(i, C, l(steps′))
            d(i, C) ⇒ F.insert(i, C, marked: false)
            f(i, C) ⇒ F.insert(i, C, marked: C = ⊥)
            r(R) ⇒
                R′ ← {(s, t) ∈ R | ∃x.(t, x) ∈ F}
                F ← F − {(t, F_t) | (s, t) ∈ R′} + {(s, F_t) | (s, t) ∈ R′}
                revcert ← revcert, r(R′)
    return revcert
```

and reading directly to disk (so the entire proof is never in memory at once). In the first pass, the elaborator reads the FRAT file and produces a temporary file (which may be stored on disk or in memory depending on configuration). The temporary file is essentially the original FRAT file with the steps put in reverse order, while satisfying the following additional conditions:

- All `a` steps have annotations.
- Every clause introduced by an `o`, `a`, or `r` step ultimately contributes to the proof of $\bot$. Note that we consider an `r` step as using an old clause with the old ID and introducing a new clause with the new ID.
- There are no `f` steps.

Algorithm 1 shows the pseudocode of the first pass, Elaborate(*cert*). Here, *cert* is the FRAT proof obtained from the SAT solver, and the pass works by iterating over its steps in reverse order, producing the temporary file *revcert*. The map $F$ maintains the active formula as a map with unique IDs for each clause (double inserts and removes to $F$ are always error conditions), and the effect of each step is replayed backwards to reconstruct the solver's state at the point each step was produced.

**Algorithm 2** Second pass (renumbering): elaborated reversed FRAT to LRAT

**function** RENUMBER($F_{\text{orig}}, revcert$)
  $M \leftarrow \emptyset,\ k \leftarrow |F_{\text{orig}}|,\ lrat \leftarrow []$    ▷ $M$ is a map ID → ID
  **for** *step* **in** reverse($revcert$) **do**
    **case** *step* **of**
      `o`$(i, C) \Rightarrow$ find $j$ such that $C \simeq (F_{\text{orig}})_j$; $M$.insert$(i, j)$
      `a`$(i, C,$ `l`$(steps)) \Rightarrow$
        $k \leftarrow k + 1$; $M$.insert$(i, k)$
        $lrat \leftarrow lrat,$ `add`$(k, C, [\pm M_i \mid \pm i \in steps])$
        **if** $C = \bot$ **then return** $lrat$
      `d`$(i, C) \Rightarrow lrat \leftarrow lrat,$ `del`$(k, M$.remove$(i))$
      `r`$(R) \Rightarrow M \leftarrow M - \{(s, M_s) \mid (s, t) \in R\} + \{(t, M_s) \mid (s, t) \in R\}$
  **assert false**    ▷ no proof of $\bot$ found

- All `d` or `f` clauses are immediately inserted to $F$, but (with the exception of the empty clause) are marked as not necessarily required for the proof, and the `d` step is deferred until just before its first use (or rather, just after the last use).
- PROVERAT($F, C$), not given here, checks that $C$ has property RAT with respect to $F$, and produces a step list in LRAT format (where positive numbers are clause references in a unit propagation proof, and negative numbers are used in RAT steps, indicating the clauses to resolve against).
- CHECKHINT($F, C, steps$) does the same thing, but it has been given a candidate proof, *steps*. It will check that *steps* is a valid proof, and if so, returns it, but the steps in the unit propagation proof may be out of order (in which case they are reordered to LRAT conformity), and if the given proof is not salvageable, it falls back on PROVERAT($F, C$) to construct the proof.

In the second pass, RENUMBER($F_{\text{orig}}, revcert$) reads the input DIMACS file and the temporary file from the first pass, and produces the final result in LRAT format. Not much checking happens in this pass, but we ensure that the `o` steps in the FRAT file actually appear (up to permutation) in the input. The state that is maintained in this pass is a list of all active clause IDs, and the corresponding list of LRAT IDs (in which original steps are always numbered sequentially in the file, and add/delete steps use a monotonic counter that is incremented on each addition step).

The resulting LRAT file can then be verified by any of the verified LRAT checkers [26] (and our toolchain also includes a built-in LRAT checker for verification).

The 2-pass algorithm is used in order to optimize memory usage. The result of the first pass is streamed out so that the intermediate elaboration result does not have to be stored in memory simultaneously. Once the temporary file is streamed out, we need at least one more pass to reverse it (even if the labels did not need renumbering) since its steps are in reverse order.

## 5 Test results

We performed benchmarks comparing our FRAT toolchain (modified CaDiCaL + FRAT-to-LRAT elaborator written in Rust) against the DRAT toolchain (standard CaDiCaL + DRAT-trim) and measured their execution times, output file sizes, and peak memory usages while solving SAT instances in the DIMACS format and producing their LRAT proofs. All tests were performed on Amazon EC2 `r5a.xlarge` instances, running Ubuntu Server 20.04 LTS on 2.5 GHz AMD EPYC 7000 processors with 32 GB RAM and 512 GB SSD.

The instances used in the benchmark were chosen by selecting all 97 instances for which default-mode CaDiCaL returned 'UNSAT' in the 2019 SAT Race results. One of these instances was excluded because DRAT-trim exhausted the available 32GB memory and failed during elaboration. Although this instance was not used for comparisons below, we note that it offers further evidence of the FRAT toolchain's efficient use of memory, since the FRAT-to-LRAT elaboration of this instance succeeded on the same system. The remaining 96 instances were used for performance comparison of the two toolchains. [4]

Figures 5 and 6 show the time and memory measurements from the benchmark. We can see from Figure 5 that the FRAT toolchain is significantly faster than DRAT toolchain. Although the modified CaDiCaL tends to be slightly (6%) slower than standard CaDiCaL, that overhead is more than compensated by a median 84% decrease in elaboration time (the sum over all instances are 1700.47 s in the DRAT toolchain vs. 381.70 s in the FRAT toolchain, so the average is down by 77%). If we include the time of the respective solvers, the FRAT + modified CaDiCaL toolchain takes 53.6% of the DRAT + CaDiCaL toolchain on median. The difference in the toolchains' time budgets is clear: the DRAT toolchain spends 42% of its time in solving and 58% in elaboration, while FRAT spends 85% on solving and only 15% on elaboration.

Figure 6 shows a dramatic difference in peak memory usage between the FRAT and DRAT toolchains. On median, the FRAT toolchain used only 5.4% as much peak memory as DRAT. (The average is 318.62 MB, which is 11.98% that of the DRAT toolchain's 2659.07 MB, but this is dominated by the really large instances. The maximum memory usage was 2.99 GB for FRAT and 21.5 GB for DRAT, but one instance exhausted the available 32 GB in DRAT and is not included in this figure.) This result is in agreement with our initial expectations: the FRAT toolchain's 2-pass elaboration method allows it to limit the number of clauses held in memory to the size of the active set used by the solver, whereas the DRAT toolchain loads all clauses in a DRAT file into memory at once during elaboration. This difference suggests that the FRAT toolchain can be used to verify instances that would otherwise require more memory than the system limit on the DRAT toolchain.

There were no noticeable differences in the sizes or verification times of LRAT proofs produced by the two toolchains. On average, LRAT proofs produced by

[4] A CSV of detailed benchmark results can be found at https://github.com/digama0/frat/blob/tacas/benchmark/benchmark-results.csv.

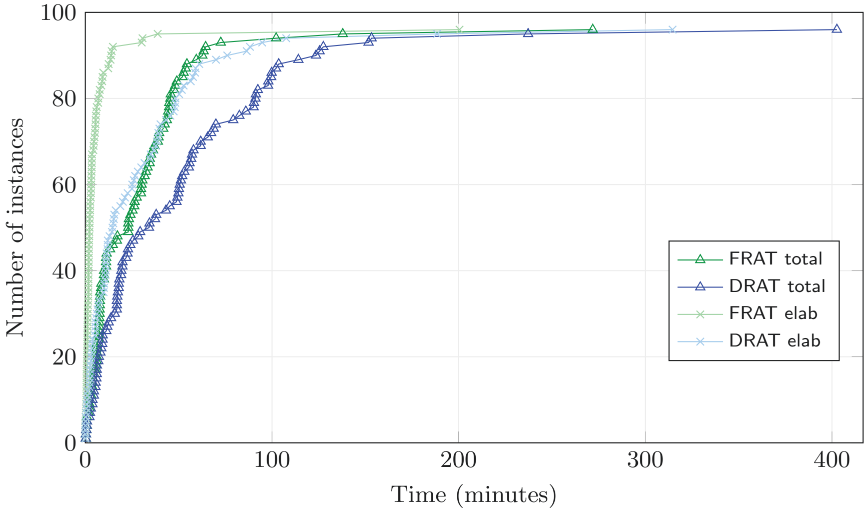

**Fig. 5.** FRAT vs. DRAT time comparison. The datapoints of 'FRAT total' and 'DRAT total' show the number of instances that each toolchain could generate LRAT proofs for within the given time limit. The datapoints of 'FRAT elab' and 'DRAT elab' show the number of instances whose intermediate format proof files (FRAT or DRAT) could be elaborated to LRAT within the given time limit.

the FRAT toolchain were 1.873% smaller and 3.314% faster[5] to check than those from the DRAT toolchain.

One minor downside of the FRAT toolchain is that it requires the storage of a temporary file during elaboration, but we do not expect this to be a problem in practice since the temporary file is typically much smaller than either the FRAT or LRAT file. In our test cases, the average temporary file size was 28.68% and 47.60% that of FRAT and LRAT files, respectively. In addition, users can run the elaborator with the `-m` option to bypass temporary files and write the temporary data to memory instead, which further improves performance but foregoes the memory conservation that comes with 2-pass elaboration.

The CaDiCaL modification is only a prototype, and some of its weaknesses show in the data. The general pattern we observed is that on problems for which the predicted CaDiCaL code paths were taken, the generated files have a large number of hints and the elaboration time is negligible (the "FRAT elab" line in fig. 5); but on problems which make use of the more unusual in-processing operations, many steps with no hints are given to the elaborator, and performance becomes comparable to DRAT-trim. For solver developers, this means that there

[5] One instance was omitted from the LRAT verification time comparison due to what seems to be a bug in the standard LRAT checker included in DRAT-trim. Detailed information regarding this instance can be found at https://github.com/digama0/frat/blob/tacas/benchmark/README.md.

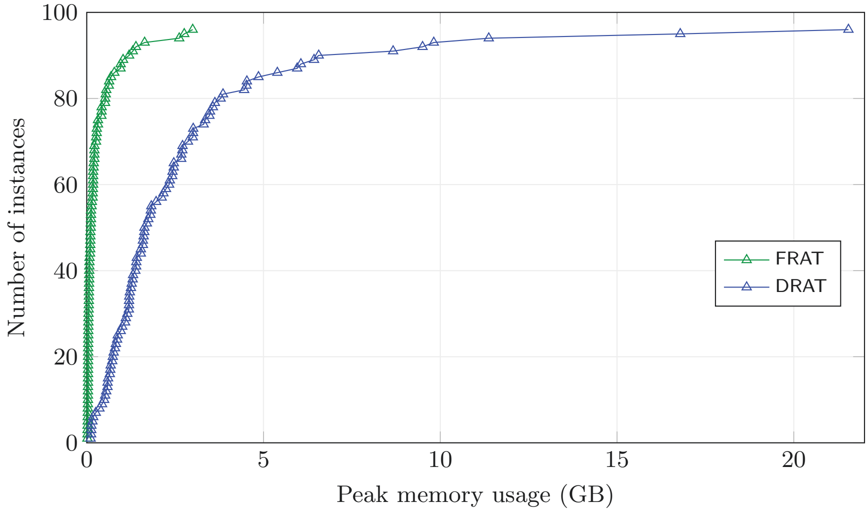

**Fig. 6.** FRAT vs. DRAT peak memory usage comparison. Each datapoint shows the number of instances that each toolchain could successfully generate LRAT proofs for within the given peak memory usage limit.

is a very direct relationship between proof annotation effort and mean solution + elaboration time. Currently, elaboration of FRAT files with no annotations (the worst-case scenario for the FRAT toolchain) typically takes slightly more than twice as long as elaboration of DRAT files with DRAT-trim, likely due to missing optimizations from DRAT-trim that could be incorporated, but this only underscores the effectiveness of adding hints to the format.

## 6 Related works

As already mentioned, the FRAT format is most closely related to the DRAT format [8], which it seeks to replace as an intermediate output format for SAT solvers. It is also dependent on the LRAT format and related tools [3], as the FRAT toolchain targets LRAT as the final output format.

The GRAT format [16] and toolchain also aims to improve elaboration of SAT unsatisfiability proofs, but takes a different approach from that of FRAT. It retains DRAT as the intermediate format, but uses parallel processing and targets a new final format with more information than LRAT in order to improve overall performance. GRAT also comes with its own verified checker [17].

Specifying and verifying the program correctness of SAT solvers (sometimes called the *autarkic* method, as opposed to the proof-producing *skeptical* method) is a radically different approach to ensuring the correctness of SAT solvers. There have been various efforts to verify nontrivial SAT solvers [18,20,19,4,5]. Although these solvers have become significantly faster, they cannot compete with the

(unverified) state-of-the-art solvers. It is also difficult to maintain and modify certified solvers. Proving the correctness of nontrivial SAT solvers can provide new insights about key invariants underlying the used techniques [5].

Generally speaking, devising proof formats for automated reasoning tools and augmenting the tools with proof output capability is an active research area. Notable examples outside SAT solving include the LFSC format for SMT solving [23] and the TSTP format for classical first-order ATPs [24]. In particular, the recent work on the VERIT SMT solver [1] is motivated by similar rationales as that for the FRAT toolchain; the key insight is that a proof production pipeline is often easier to optimize on the solver side than on the elaborator side, as the former has direct access to many types of useful information.

## 7 Conclusion

The test results show that the FRAT format and toolchain made significant performance gains relative to their DRAT equivalents in both elaboration time and memory usage. We take this as confirmation of our initial conjectures that (1) there is a large amount of useful and easily extracted information in SAT solvers that is left untapped by DRAT proofs, and (2) the use of streaming verification is the key to verifying very large proofs that cannot be held in memory at once.

The practical ramification is that, provided that solvers produce well-annotated FRAT proofs, the elaborator is no longer a bottleneck in the pipeline. Typically, when DRAT-trim hangs it does so either by taking excessive time, or by attempting to read in an entire proof file at once and exhausting memory (the so-called "uncheckable" proofs that can be produced but not verified). But FRAT-to-LRAT elaboration is typically faster than FRAT production, and the memory consumption of the FRAT-to-LRAT elaborator at any given point is proportional to the memory used by the solver at the same point in the proof. Since LRAT verification is already efficient, the only remaining limiting factor is essentially the time and memory usage of the solver itself.

In addition to performance, the other main consideration in the design of the FRAT format and toolchain was flexibility of use and extension. The encoding of FRAT files allows them to be read and parsed both backward and forward, and the format can be modified to include more advanced inferences, as we have discussed in the example of XOR steps. The optional `l` steps allow SAT solvers to decide precisely when they will provide explicit proofs, thereby promoting a workable compromise between implementation complexity and runtime efficiency. SAT solver developers can begin using the format by producing the most bare-bones FRAT proofs with no annotations (essentially DRAT proofs with metadata for original/final clauses) and gradually work toward providing more complete hints. We hope that this combination of efficiency and flexibility will motivate performance-minded SAT solver developers to adopt the format and support more robust proof production, which is presently only an afterthought in most SAT solvers.
